# Mesenchymal Stromal Cells Derived from Canine Adipose Tissue: Evaluation of the Effect of Different Shipping Vehicles Used for Clinical Administration

**DOI:** 10.3390/ijms25063426

**Published:** 2024-03-18

**Authors:** Valentina Andreoli, Priscilla Berni, Virna Conti, Roberto Ramoni, Giuseppina Basini, Stefano Grolli

**Affiliations:** Department of Veterinary Science, University of Parma, 43126 Parma, Italy; priscilla.berni@unipr.it (P.B.); virna.conti@unipr.it (V.C.); roberto.ramoni@unipr.it (R.R.); giuseppina.basini@unipr.it (G.B.)

**Keywords:** mesenchymal stromal cells, cell shipment, storage vehicle, clinical administration, dog, veterinary regenerative medicine

## Abstract

Mesenchymal Stromal Cells (MSCs)-based therapies are rapidly gaining interest in veterinary medicine. Cellular therapy represents a new challenge for practitioners and requires precise coordination between the cell processing laboratory and the veterinary clinic. Cryopreservation is the best method to provide fast, in-time, and long-distance delivery of cells for therapeutic applications. However, potentially toxic cryoprotectants and xenobiotic products make the direct administration of cells impracticable for patients. Alternatively, the cells may be resuspended in a ready-to-use vehicle and shipped to the veterinary clinic. In this study, two nutrient-poor vehicles (physiologic saline and ringer lactate solutions) and two nutrient-rich vehicles (the releasate derived from autologous Platelet Poor Plasma and Platelet Rich Plasma) were tested on adipose tissue-derived canine MSCs (AD-MSCs). AD-MSCs stored for 2, 4, or 24 h in the different media were compared regarding mortality, metabolic activity, and replicative capacity. Furthermore, antioxidant activity and the pattern of expression of genes related to AD-MSCs function were performed following 24 h of storage. The results showed that all the different vehicles preserve cell vitality and replication following short-term storage. In long-term storage, the vehicle and cell density affect cell vitality, proliferation, and gene expression (CCL-2, CXCR-4, and TSG-6). Nutrient-rich vehicles seem better suited to preserve cell functionalities in this contest.

## 1. Introduction

Regenerative medicine based on cell administration is increasingly considered a therapeutic option for various diseases in veterinary patients [1]. In particular, Mesenchymal Stromal Cells (MSCs) play a prominent role in developing cell treatments to reinforce or replace traditional therapeutic approaches. MSCs have been isolated from many adult tissues and extensively studied for their peculiar therapeutic potential in different animal species. The dog is one of the species in which cell therapies are more actively studied and used [2]. MSCs have found a relevant place in cell therapies based not only on their regenerative potential but also on their immunomodulatory and angiogenic properties, making them suitable for application not only in musculoskeletal disorders, where the local application is privileged, but also in disorders that require a systemic administration (i.e., immune-mediated diseases, cardiovascular diseases and organ pathologies) [3,4,5].

The increasing use of MSCs-based therapies in veterinary medicine draws attention to important issues related to potential logistic bottlenecks throughout the whole process of isolation, expansion, handling, and administration of MSCs. Every step in the process should be analyzed to assess how it can influence MSC’s therapeutic features [6,7,8,9].

The interface between the veterinary surgeon and the laboratory responsible for the cell preparation represents one of these critical points. Challenging steps involving the practitioner’s responsibility remain; for example, the collection, management, and delivery of the starting tissue sample to the laboratory and the correct strategy for cell delivery from the laboratory to the veterinary clinic for their administration to the patient. Being a biological product sensitive to environmental conditions (i.e., temperature, mechanical stress, etc.), MSCs, if not adequately handled and stored immediately before their administration, could suffer stress and damages ranging from increased mortality to impaired therapeutic functions [10,11]. In particular, carefully evaluating shipping and administration modalities is essential since they can potentially compromise cells’ biological and therapeutic activity [9]. A typical shipping method of MSC preparation involves the cryopreservation of cells in appropriate media and their administration to the patient after thawing and removing the xenobiotic and cryopreserving components [12,13]. This procedure requires the clinician to properly manage the cryopreserved material upon arrival, ensuring both the preparation’s safety and the cells’ quality. The management of MSCs in this context requires adequate instrumentation and training of the staff and exposes the patient to greater risk, given the possible residual traces of cryopreserving components and microbial contamination due to incorrect handling [6,14]. For these reasons, the shipping of cryopreserved cells is avoided for short-term transport (usually within 24 h) by privileging the shipment of MSCs already suspended in a medium, which are then exploited for local or systemic administration [15].

When prepared for clinical application, MSCs are either collected directly from culture plates (freshly cultured cells) or, alternatively, they are preserved and stored in liquid nitrogen (cryopreserved cells) and then thawed and resuspended immediately before administration [9,10,16]. Both procedures are used in veterinary medical practice. When the cell preparation laboratory is close to the clinic, cells can be collected from the culture vessel or thawed, resuspended in the appropriate vehicle, and applied following short-term storage at room temperature. Conversely, cell delivery may take some time when the manufacturing laboratory is far from the clinic. In this case, the cells would have to be transported and maintained for hours in a suitable preservation medium at room temperature or 4 °C [11,17].

The present study aimed to evaluate the short- (two and four hours) and long-term (twenty-four hours) effects of some of the most common nutrient-poor and nutrient-rich vehicles used for the clinical administration of canine adipose tissue-derived MSCs (AD-MSCs). Cell storage was tested at room temperature, evaluating the effect on the potency and vitality of MSCs. Vehicles considered in the study were chosen among the most used in clinical applications according to the published literature for dogs and horses: physiological saline solution (0.9% NaCl *w*/*v*) [18,19,20], Ringer’s Lactate saline solution (RLS) [21] (nutrient-poor vehicles), Platelet Rich Plasma (PRP) [22,23], and Platelet Poor Plasma (PPP) [24] (nutrient-rich vehicles). The DMEM (Dulbecco’s Modified Eagle’s Medium) culture medium, supplemented with 10% fetal bovine serum (FBS), was used as a control. Cells parameters assessed at different time intervals included viability, replicative capacity, non-enzymatic anti-oxidant activity, and analysis of the expression of a set of genes involved in the biological activity of MSCs using Real-Time PCR.

## 2. Results

### 2.1. Phenotypic Characterization and Differentiation of AD-MSCs

**Figure 1:**
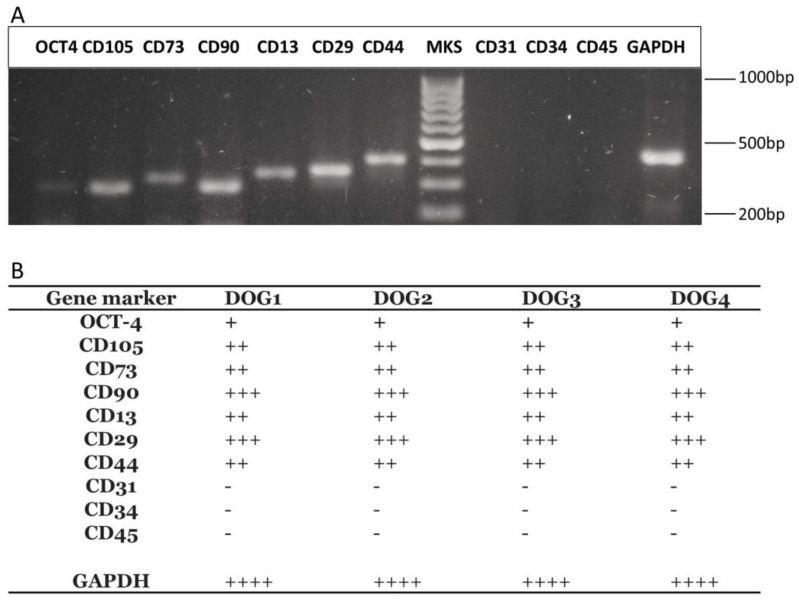
Phenotypic characterization of AD-MSCs. Gene expression analysis of AD-MSCs markers by RT-PCR. (**A**) Representative agarose gel picture of the expression of markers used for the characterization of the AD-MSCs populations, relative to Dog1. (**B**) Panel of gene expression of the different AD-MSCs populations evaluated. Legend: - (not expressed); + (<25% of GAPDH signal); ++ (25–50% of GAPDH signal); +++ (>50% of GAPDH signal); ++++ (≥100% of GAPDH signal).

**Figure 2:**
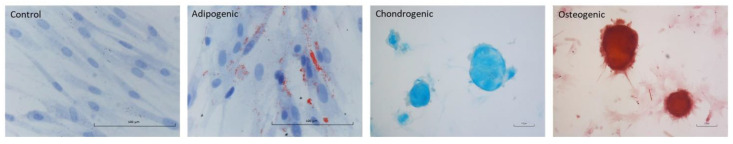
AD-MSCs were able to differentiate into osteogenic, adipogenic, and chondrogenic lineages when induced with a specific differentiation medium. Bars = 100 µm.

The phenotypic characterization of AD-MSCs performed by RT-PCR showed cell positivity to the following markers, typical of MSCs: CD90, CD73, CD105, CD44, CD13, CD29, (Figure 1A,B) [19,25]. The cells were negative to the expression of CD45, CD34, and CD31, confirming the previously validated gene expression panel [26,27]. No significant difference was observed between the different cell preparations. The ability of the AD-MSCs to differentiate towards osteogenic, chondrogenic, and osteogenic lineages was confirmed for P3 cultures after applying the corresponding induction media (Figure 2).

### 2.2. Effects of Different Vehicles on Cells Stored for 2 and 4 h

**Figure 3:**
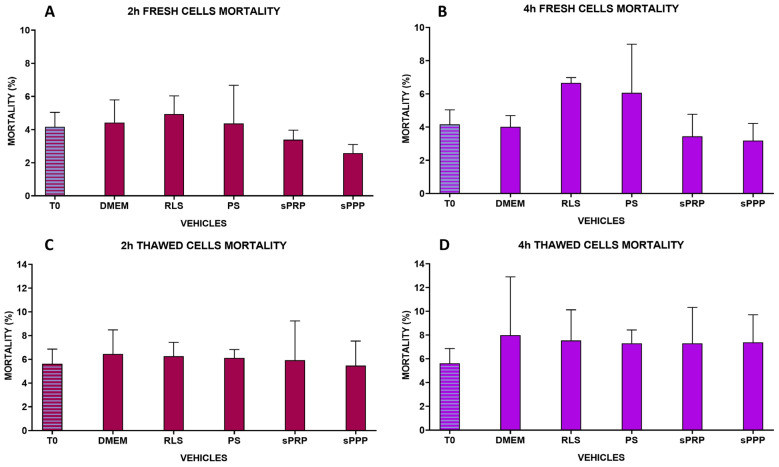
Effects of different vehicles on cell viability following 2 (**A**,**C**) and 4 (**B**,**D**) hours storage at room temperature, on fresh MSCs (fMSCs) (**A**,**B**) and frozen/thawed MSCs (tMSCs) (**C**,**D**). Data are reported as mean mortality rate for each time point and vehicle.

Figure 3 shows the viability of AD-MSCs kept for 2 and 4 h at room temperature in the five different vehicles evaluated. The mortality rate, evaluated by Trypan blue staining, was not affected by the different environments of storage. Furthermore, the mortality rate of cells stored in the different vehicles was not different concerning the mortality evaluated just after fresh cells (fMSCs) collection by trypsinization (Figure 3A,B) or stored cells (tMSCs) thawing (Figure 3C,D). The mean mortality rate was below 10% for each vehicle and time evaluated. Following treatment with the different cell vehicles, cell morphology was evaluated after seeding AD-MSCs into culture plates. Explanatory images are visible in the Supplementary Materials (Figure S1). No differences were observed after 2 or 4 h of cell storage in the different vehicles.

**Figure 4:**
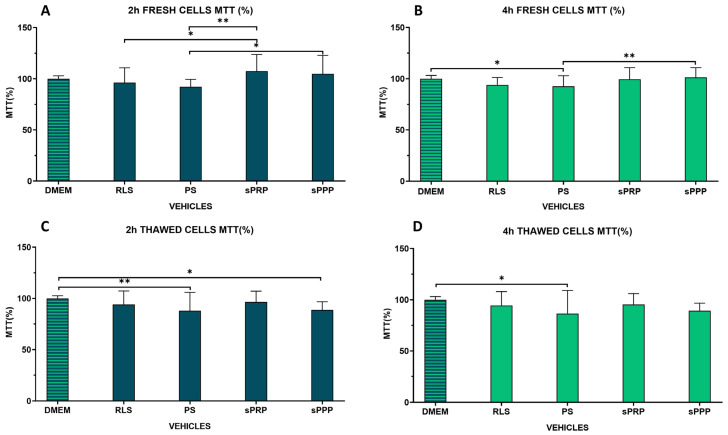
Effects of different carrier solutions on MTT assay following 2 (**A**,**C**) and 4 (**B**,**D**) hours storage at room temperature, on fresh MSCs (fMSCs) (**A**,**B**) and frozen/thawed MSCs (tMSCs) (**C**,**D**). Data are expressed as a percentage of control cultures maintained in DMEM. DMEM: control medium, RLS: Ringer Lactate Solution; PS: physiological Saline; sPRP: Platelet Rich Plasma; sPPP: Platelet Poor Plasma; * *p* < 0.05, ** *p* < 0.01. The brackets indicate binary comparison between groups showing statistically significant differences.

The metabolic activity of AD-MSCs was evaluated by MTT assay (Figure 4). The statistical analysis highlights the presence of significant differences between the various vehicles, even if, from a quantitative point of view, the differences are rather limited. In particular, for fresh cell preparations (fMSCs) after storage of 2 h (Figure 4A), a lower metabolic activity was observed in cells stored in physiological saline (PS) in comparison to sPRP (*p* < 0.01) and sPPP (*p* < 0.05). Ringer lactate solution (RLS) was inferior to sPRP (*p* < 0.05). No vehicle analyzed was found to be inferior to DMEM control. After a 4 h storage (Figure 4B) PS demonstrated a lower metabolic activity with respect to DMEM control (*p* < 0.05) and sPPP (*p* < 0.01). With regard to frozen/thawed cells (tMSCs) at 2 h storage, sPPP (*p* < 0.05) and PS (*p* < 0.01) vehicles were inferior to DMEM control (Figure 4C). At 4 h, only PS (*p* < 0.01) demonstrated an absorbance signal inferior to DMEM control (Figure 4D). Fresh cells stored in nutrient-rich vehicles demonstrated slightly higher metabolic activity with respect to nutrient-poor vehicles (*p* < 0.05).

### 2.3. Effects of Different Vehicles on Cells Stored for 24 h in Different Carrier Solutions

**Figure 5:**
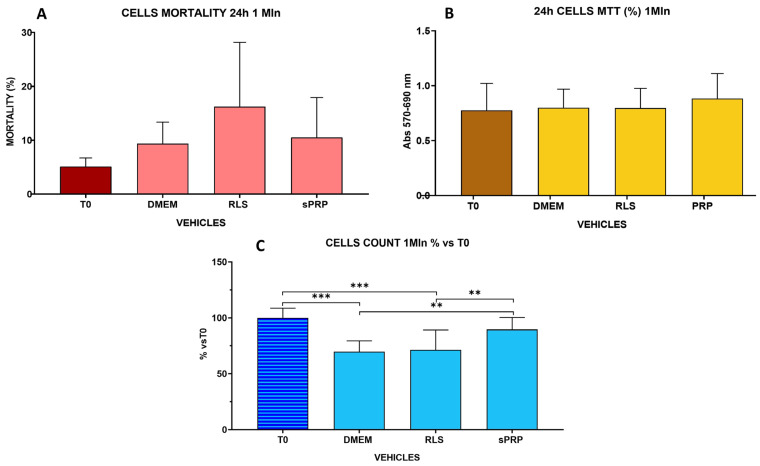
Effect of different vehicles on fresh MSCs storage at room temperature for 24 h. Mortality rate (**A**), metabolic activity by MTT assay (**B**), and cell proliferation by direct cell count (**C**) were evaluated for Ringer Lactate Solution (RLS), sPRP, and DMEM. T0 represents the results for MSCs directly collected from the culture plates, without the storage period. DMEM: control medium, RLS: Ringer Lactate Solution; sPRP: Platelet Rich Plasma. ** *p* < 0.01, *** *p* < 0.001. The brackets indicate binary comparison between groups showing statistically significant differences.

This set of experiments was done to analyze the effects of the different vehicles on the storage of fresh MSCs at room temperature for 24 h, simulating a clinical situation involving the preservation and/or transport of cells for a significant number of hours. The analyzed vehicles were RLS and sPRP. DMEM supplemented with 10% FBS was used as a reference storage vehicle. The three different vehicles were compared with the results obtained evaluating the different parameters in MSCs directly collected from the culture plates, without storage (T0). MSC mortality rate, cell metabolic activity, and replication rate evaluated by direct cell count were used to assess the effect of cell storage using 1 mL of 1 × 10^6^ AD-MSCs/mL (Figure 5).

Figure 5A reports the percentage of cell mortality. No statistically significant difference was observed between the three vehicles although the mortality rate was higher following cell storage in RLS. In particular, at T0 the cells exhibited an average mortality rate of 5.1 ± 1.6%. Cells maintained in DMEM for 24 h showed an average mortality rate of 9.4 ± 3.9%; cells in sPRP reported an average mortality rate of 10.5 ± 7.4%, while cells in RLS exhibited an average mortality rate of 16.23 ± 11.9%. No statistically significant differences were observed comparing the vehicles to T0 control cultures.

With regard to the metabolic activity, evaluated by the MTT test, and the replicative activity measured by direct cell count (Figure 5B,C), a difference was observed between the vehicles. In particular, sPRP demonstrated a higher replication rate with respect to RLS (*p* < 0.01), with no differences when compared to T0 cells (Figure 5C). MTT test did not show a statistically significant difference between sPRP and RLS (Figure 5B).

**Figure 6:**
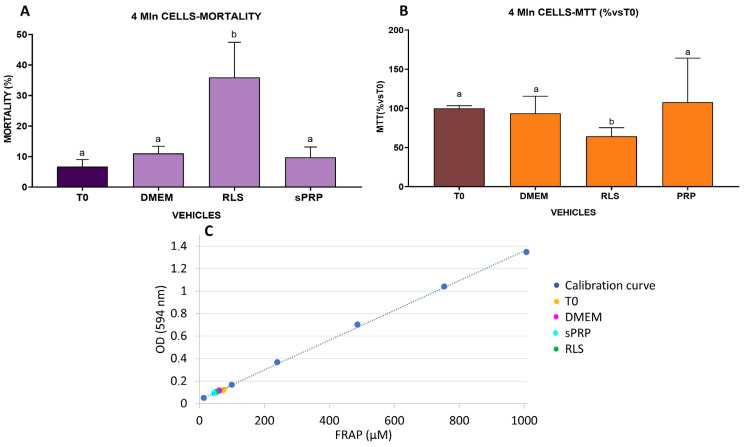
Effects of different carrier solutions on mortality (**A**) and metabolic activity measured by MTT assay (**B**) of AD-MSCs stored for 24 h at room temperature in RLS and sPRP. Cells stored in RLS showed a higher mortality rate ((**A**), *p* < 0.001) and a lower metabolic activity ((**B**), *p* < 0.001) in comparison to the other vehicles and MSCs directly collected from the culture plates without the storage period (T0). The ferric reducing ability (FRAP assay) (**C**) did not show differences between AD-MSCs stored in the different vehicles. Different letters on the bars indicate a significant difference (*p* < 0.001).

A further set of experiments was run storing 4 mL of 1 × 10^6^ AD-MSCs/mL for 24 h at room temperature, thus evaluating the effects of the vehicles on a higher number of cells and larger volumes. Statistical analysis highlighted that in this case, AD-MSCs behavior was different when stored in RLS (Figure 6). The cell mortality rate was higher (*p* < 0.0001) in this vehicle with an average of died cells 30 percentage points higher than T0 cells and all other vehicles (Figure 6A). Following the treatment with the different cell vehicles, cell morphology was evaluated after seeding into culture plates. Explanatory images are visible as Supplementary Materials (Figure S2). A higher rate of non-attached cells was observed when AD-MSCs were stored for 24 h in RLS, reflecting the higher number of dead cells counted by the Trypan Blue assay.

The marked mortality of cells stored in RLS was also reflected by the analysis of metabolic activity by the MTT test: in this case, the metabolic activity of cells stored in RLS was significantly lower when compared to the other vehicles (*p* < 0.0001) (Figure 6B).

Finally, the FRAP test performed on these same cell populations for the evaluation of the non-enzymatic antioxidant activity did not show any differences between the vehicles and the T0 (Figure 6C).

### 2.4. Gene Expression Analysis

**Figure 7:**
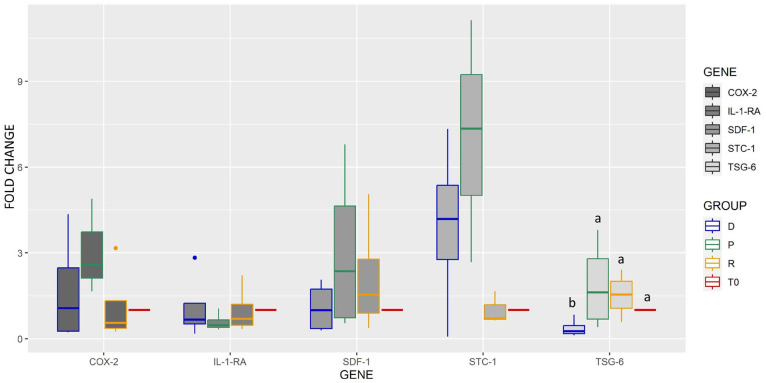
Box plot (median, quartiles) representing the expression fold change (log2) of COX-2, IL1Ra, SDF-1, STC-1, and TSG-6 after 24 h of cell exposure in different vehicles (D = DMEM control vehicle, P = sPRP, R = Ringer Lactate Solution), compared to freshly cultured cells (T0). Single outliers are indicated as spots. A significant difference marked with letters (a, b) was observed for TSG-6, downregulated in the control vehicle (*p* < 0.05).

**Figure 8:**
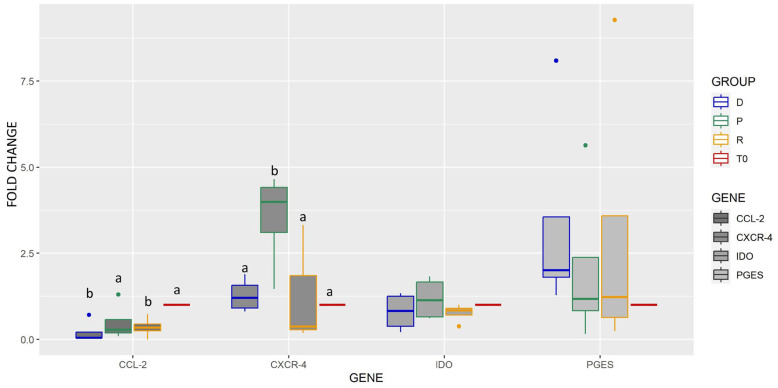
Box plot (median, quartiles) representing the expression fold change (log2) of CCL-2, CXCR-4, IDO, and PGES after 24 h of cell exposure in different vehicles (D = DMEM control vehicle, P = sPRP, R = Ringer Lactate Solution), compared to freshly cultured cells (T0). Single outliers are indicated as spots. A significant difference marked with letters (a, b) was observed for CCL-2, downregulated in the DMEM control vehicle and the Ringer group, while CXCR-4 was upregulated in the sPRP group (*p* < 0.05).

Real-time quantitative PCR was used to compare the expression of some genes involved in the immunomodulatory properties of MSCs after exposure to different vehicles for 24 h at room temperature and compared with cells analyzed immediately after collection from culture plates (T0 cells) (Figure 7 and Figure 8). Statistically significant changes were observed for CCL2, TSG-6, and CXCR4 gene expression. In detail, TSG-6 expression was lower in the control group (*p* < 0.05) (Figure 7).

CCL2 expression was lower in RLS and control groups, while CXCR4 expression was higher in the sPRP group (*p* < 0.05) (Figure 8). No significant difference was observed in the expression of STC-1, COX-2, IL1RA, SDF-1, PGES and IDO.

## 3. Discussion

MSCs hold great potential in veterinary regenerative medicine. The recent scientific literature supports the feasibility and safety of delivering stem cells in a clinical setting, thus justifying the expansion of their clinical use in recent years [28]. Setting up a proper protocol for cells’ transportation to the administration site is one of the most delicate phases in managing MSC therapy. Some recent works have highlighted how MSCs immediately after thawing demonstrate less effective results in in vitro potency assay when compared to cells maintained in culture, particularly regarding immuno-modulatory properties [29,30]. On the contrary, other studies suggest that cryopreserved MSCs maintain proper cell viability and potency post-thawing [2,9,11].

An important practical drawback of cryopreservation is that MSCs must be manipulated to remove cryoprotectant and xenobiotic agents before administration. This procedure requires the receiving veterinary clinic to properly handle the material upon arrival to avoid contamination and/or loss of biological activity of the cell product. For these reasons, the delivery of cryopreserved cells is often avoided in case of short-term transport. In these cases, cells are prepared in a specialized laboratory and sent to the practitioner already resuspended in appropriate vehicles for clinical application.

In this scenario, some published papers assessed the effects of different vehicles commonly used for cell administration, investigating different times and temperature ranges, such as room temperature, refrigeration temperature (4–8 °C), or cell cultures’ incubation temperature (37 °C) [3,6,12,14,17].

Bronzini et al. [17] evaluated the effects of different vehicles, times, and temperatures on canine adipose tissue-derived MSCs and horse blood-derived MSCs. The different media (PBS, DMEM) were analyzed with or without the addition of fetal bovine or horse serum and did not show substantial differences. Conversely, both temperature and storage time had a significant effect. The best survival was observed for cells stored at room temperature no later than 9–12 h, both for dog AD-MSCs and horse blood-derived MSCs. Other works report the results of equine MSC storage at room and refrigeration temperatures. Mercati et al. [31] reported that RT storage is inferior to refrigeration temperature (4 °C) for the storage of equine AD-MSCs in a DMEM culture medium. Garvican et al. [32] evaluated different storage media (serum, heparinized plasma, PRP, hyaluronic acid, isotonic saline), concluding that any of the tested storage media are adequate when bone marrow-derived equine MSCs are stored at 4–8 °C and used within 24 h. Cell death was higher in bone marrow aspirate, PRP, and serum at longer times than in isotonic saline. Espina et al. [33] evaluated a set of storage media, concluding that, consistent with other studies, the declining viability of unfrozen MSCs is observed exceeding 24 h. In a recent work by Iacono et al. [34], cells isolated from horse adipose tissue and Wharton’s jelly-derived MSCs were evaluated after storage in saline or plasma at room temperature, 4 °C and for times ranging from 6 to 48 h, concluding that for cells of adipose origin, the use of plasma gives better results and that the best conditions are maintained for cells stored no later than 24 h. For Wharton’s jelly-derived MSCs, 6 h of storage already led to apparent biological modifications.

Recently, Sultana et al. [11] evaluated different vehicles usable as carriers for the administration of canine MSCs (i.e., 0.9% saline, 5% dextrose solution, heparin in saline, Hartmann’s solution). The results showed an evident reduction in cell viability and proliferation after 12 h of storage at 4 °C. Different media also varied the expression of stemness genes and chondrogenic differentiation. The authors suggested that Ad-MSCs should be administered to the patient within 6–12 h.

Given the crucial role of the cell storage vehicle before administration, this study aimed to determine the effect on the biological activity of the MSCs population derived from canine adipose tissue of some vehicles frequently used for their transport and administration. Nutrient-poor vehicles (physiological saline (0.9% NaCl), Ringer Lactate Solution (RLS)), and nutrient-rich vehicles (Platelet Rich Plasma and Platelet Poor Plasma) were analyzed by evaluating alternative storage times (2, 4, and 24 h) at room temperature (22–26 °C). DMEM supplemented with 10% FBS was used as a control since it is one of the most common mediums used to expand canine MSCs in vitro, although unsuitable for clinical administration. Physiological saline and RLS are readily available to veterinary surgeons. PRP is a platelet concentrate rich in bioactive molecules [16,35,36] whose use is spreading in the veterinary clinic, sometimes in therapeutic applications associated with MSCs. PPP can be considered a suitable vehicle for MSC delivery, as it is easily prepared in the veterinary clinic. Both PRP and PPP can be prepared as autologous vehicles from the patient’s blood. In recent years, platelet lysate (PL) and platelet releasate (sPRP) prepared from platelet concentrates have been proposed as xeno-free supplements for MSCs expansion [35,36]. Unfortunately, the availability of canine PL and sPRP is limited, and the current literature does not fully support their use in replacement for FBS [35]. The storage of MSCs in PRP or PPP before cell administration could be considered a short-term transition step preceding the clinical administration to reduce risks associated with using FBS for cell expansion.

We decided to evaluate the effect of the different cell vehicles at room temperature (i.e., a temperature ranging between 22 and 26 °C) and short time storage (2 and 4 h) based on a procedure that can be applied when the laboratory preparing the cells and the clinics are located nearby or at a limited distance. In this case, the time interval between the cells’ preparation and administration will likely be short, depending on the patient’s preparation (sedation, anesthesia, etc.) A cell storage time of 24 h has been compared to short-time storage to evaluate the possible effects of a long-time permanence of cells in the different vehicles. This scenario could apply when clinical or organizational needs of the veterinary clinic require a shipment to significant distances or cause a delay in their administration. The AD-MSCs used in the present study were prepared using a protocol previously described by us [26] and widely used in the literature. Based on the work of Ivanovska et al., phenotypic characterization was performed by RT-PCR (Figure 1) using a panel of previously validated genes [26]. The cell preparations used ensure consistency with previously published results, as suggested by Guest et al. [37]. The three-lineage ability of differentiation of the AD-MSCs was confirmed after exposure to specific differentiation media (Figure 2).

The study compared fresh-cultured and cryopreserved AD-MSCs thawed just before their use. The cell viability of fresh and thawed cells did not show statistically significant differences between nutrient-poor and nutrient-rich vehicles following 2 and 4 h of storage. All the vehicles ensured a mortality rate below 10%, up to 4 h, for fresh and thawed cells. Nonetheless, the MTT assay underlined a difference between nutrient-poor (PS, RLS) and nutrient-enriched vehicles (sPRP, sPPP) and between poor vehicles and DMEM control for fresh and thawed cells. Although statistically significant, the observed differences between nutrient-poor and rich vehicles remain minimal, and it is not clear whether they could have a biological significance. Based on these results, all the storage/transport vehicles analyzed can be used in a clinical context that foresees a short storage period from resuspension of cells to administration. In particular, the results confirm that sPPP and sPRP are safe and effective vehicles for short-time storage. The subsequent experimental set-up evaluated the effects of transport vehicles for longer storage time (24 h) at room temperature. Since no major differences were highlighted in previous experiments, the experiments compared RLS (nutrient-poor) and sPRP (nutrient-rich) vehicles to DMEM storage using fresh cultured cells. The results (Figure 5A) were consistent with the evidence reported by Bronzini and colleagues [17]; cell mortality increased after 24 h of permanence in an environment other than the culture medium, particularly for cells stored in RLS. Nonetheless, the difference in mortality rate was not statistically significant for any of the vehicles. Furthermore, cell replication ability and metabolic activity were investigated by direct cell count and MTT assay in fresh AD-MSCs stored for 24 h in the different vehicles. Cell viability was not different between the vehicles, and no difference was observed compared to the DMEM control. On the contrary, the MTT assay underlined a difference between sPRP and all other vehicles (RLS, DMEM, and T0) with increased metabolic activity in AD-MSCs stored in sPRP (*p* < 0.0001). No statistically significant differences were observed in direct cell counts. The last set of experiments was conducted using larger volumes of vehicles (4 mL), keeping the concentration of 1 × 10^6^ cells/mL constant. Mortality, metabolic activity, antioxidant capacity (FRAP test), and expression of genes involved in the immunomodulatory properties of MSCs were evaluated. RLS was less suitable as a cell vehicle in these experimental conditions, demonstrating a higher mortality rate than the other vehicles tested. The same results were obtained comparing the metabolic activity: populations maintained in RLS for 24 h showed a decreased metabolic capacity compared to control, T0, and sPRP. These results lead us to hypothesize that RLS, in the presence of high numbers of cells, cannot ensure adequate viability and replicative capacity, casting doubts on its suitability to store preparations containing high numbers of cells.

The therapy for localized pathologies, such as, for example, osteoarthritis, requires numbers of cells ranging between 2 and 20 × 10^6^ cells per joint [38]. In comparison, systemic administrations use variable numbers ranging between 1.5 and 3 × 10^6^ cells per kg of animal weight [13,19,20,21]. These cell dosages indicate the importance of exploring the behavior of cells suspended in administration vehicles using adequate cell numbers to evaluate the role of different storage vehicles effectively.

The FRAP test for the detection of non-enzymatic antioxidant capacity did not detect variations among examined populations, suggesting no differences in the behavior of these cells compared to the control. To evaluate a possible interference of the storage vehicles on the activity of the AD-MSCs, the expression of a set of genes involved in the immunomodulatory activity of these cells was evaluated. The panel includes TSG-6, SDF-1, IL1Ra, STC-1, CCL-2, CXCR-4, COX-2, and PGES gene. TSG-6 and IL-1-Ra contribute to the anti-inflammatory and immunomodulating activity of MSCs [30,39]. SDF-1/CXCR4 axis is a key player in the homing of MSCs and other progenitor cells to damaged tissues [40,41]. STC-1 has a protective role against reactive oxygen species (ROS) and a potential anti-inflammatory action [39]. COX-2 and PGES are involved in the synthesis of PGE2. PGE2 secreted by MSCs is a regulator of macrophage polarization towards the M2, an anti-inflammatory phenotype protecting against exacerbated inflammation [42]. CCL-2 promotes angiogenesis and is a key factor in the migration of MSCs [43].

Statistical analysis demonstrated significant variation relative to TSG-6, CCL-2, and CXCR-4. In detail, TSG-6 expression was lower in the DMEM control group, CCL-2 was downregulated in DMEM and RLS groups, while CXCR-4 was upregulated in the sPRP group (*p* < 0.05). The variation in the expression level of these three genes could modify the immunomodulatory properties and the homing ability of MSCs, thus suggesting that different storage vehicles can affect the therapeutic potential of these cells. In conclusion, our results suggest that cells can be maintained between 2 and 4 h in both enriched and poor vehicles. Furthermore, only cells intended for resuspension in enriched vehicles should undergo permanence for up to 24 h since resuspension in vehicles poor in nutrients would not ensure the appropriate quality standards and viability of cells. The results of the study also underline how gene expression of some genes linked to the therapeutic activity of AD-MSCs can vary following exposure to different vehicles. The possibility cannot be ruled out that further differences might be linked to genes or cellular features that were not examined in this study. Further analysis is needed to investigate this topic more extensively.

The present work has some weaknesses that need to be addressed in the future. Data suggest that storing AD-MSCs in high concentrations for prolonged periods may compromise their viability. It is a crucial aspect to explore, especially for applications requiring a high number of cells; for example, systemic and organ diseases in which intravenous administration is envisaged. In the present study, adipose tissue samples were collected during elective hysterectomy; thus, only healthy female donors were enrolled. Although, to the best of our knowledge, no differences in the therapeutic potential of cells of different sexes are reported for the canine species, some differences in gene expression have been reported for other species, which could imply sex differences in the biology of MSCs. Of course, the same could apply to MSCs derived from different sources [44]. A further weak point, familiar to most of the works published on the storage of MSCs, is that these studies are limited to evaluating the mortality, replicative, and differentiative capacity of the cells. We have proposed a panel of genes whose expression could be important for effective cell therapy, being involved in immunomodulation. We know the gene panel we propose is restricted and needs improving. For example, using proteomic or RNA-seq techniques would be more informative and desirable in the future. Nevertheless, the proposed panel covers a set of genes whose role has been extensively studied in association to MSCs therapeutic properties. The therapeutic activity of MSCs is indeed mediated by a large variety of biological factors, whose role varies in different pathologies [37]. Correlating the use of different cellular vehicles with the therapeutic efficacy of cellular treatment will require a better knowledge of the MSCs’ fundamental therapeutic mechanisms and the development of adequate potency assays indicative of the actual therapeutic capabilities of the cells in different clinical contexts.

## 4. Materials and Methods

### 4.1. Ethics Statement

All the biological material used in the present study was collected in compliance with ethical regulations and animal welfare. The adipose tissue from which the cells were isolated was provided from the local veterinary hospital (University of Parma, Department of Veterinary Medical Science) as a waste material following elective surgeries (ovariohysterectomy). Platelet Rich Plasma and Platelet Poor Plasma were provided like surplus from blood processed for clinical applications. The collection of the biological material for the isolation and expansion of MSCs was approved by the Ethics Committee of the University of Parma (OPBA protocol number 122/OPBA/2018).

### 4.2. Cell Isolation and Culture

Media, supplements, and other cell culture reagents used for cell culture were from Gibco (Thermo Fisher Scientific Inc., Waltham, MA, USA) unless differently specified. The plastic labware was from VWR (Avantor, Radnor, OR, USA). A detailed list of cell culture reagents is provided in Supplementary File S1.

Abdominal visceral adipose tissue was collected from four healthy dogs of different breeds under general anesthesia during elective ovariohysterectomy at the local Veterinary Teaching Hospital. Each owner signed an informed consent before surgery. Biopsies were stored in sterile DMEM supplemented with antibiotics (see below) and processed within 2 h. Adipose tissue samples were weighed, washed in ethanol and phosphate-buffered saline, pH 7.4, and digested in mild agitation for 1 h at 37 °C with collagenase type II (0.1% *w*/*v*) prepared in DMEM. Digested samples were centrifuged for 15 min at 180× *g*, and the pellets resuspended in DMEM supplemented with 10% FBS, penicillin (100 μg/mL), streptomycin (100 μg/mL), and amphotericin B (2.5 μg/mL) (complete DMEM, cDMEM). Resuspended cells were seeded in T25 flasks and maintained at 37 °C in an atmosphere with 5% CO_2_ in cDMEM.

When the cells reached about 70–80% of confluence, they were detached using 0.05% Trypsin-EDTA. Cells were then reseeded at 5000 cells cm^2^ and expanded until P3-P4, when they were used for the experiments. When needed, cells were cryopreserved (5 × 10^6^/vials) in liquid nitrogen using a freezing medium consisting of 50% (*v*/*v*) FBS, 10% (*v*/*v*) dimethyl sulfoxide, and 40% DMEM.

### 4.3. AD-MSCs Differentiation

To evaluate the three lineages differentiation ability of AD-MSCs, cells at P3 were plated in six-well plates at a density of 6 × 10^3^ cells/cm^2^. When cell cultures reached a confluency of 70–80% they were treated with specific reagents to induce adipogenic, chondrogenic, and osteogenic differentiation. Adipogenic and chondrogenic differentiation was induced with StemPro Adipogenesis Differentiation Kit (Gibco Thermo Fisher Scientific Inc., Waltham, MA, USA) and Stem Pro Chondrogenic Differentiation kit (Gibco Thermo Fisher Scientific Inc., Waltham, MA, USA), respectively, following the protocols provided by the producer. After 21 days the cells were fixed with 70% ethanol and stained with Oil Red O stain (Sigma-Aldrich, Inc., St. Louis, MO, USA) to evaluate the adipogenic differentiation. For the chondrogenic differentiation, the cells were fixed with 4% formaldehyde following the 21 days treatment and stained with Alcian blue (Sigma-Aldrich, Inc., St. Louis, MO, USA). For the osteogenic differentiation, P3 AD-MSCs were seeded in six-well plates at a density of 6 × 10^3^ cells/cm^2^. When cultures arrived at a confluency of about 80%, the cells were treated with osteogenic induction medium constituted by DMEM added with 100 nM dexamethasone, 10 μM glycerophosphate, and 0.250 mM ascorbic acid (all reagents by Sigma-Aldrich, Inc., St. Louis, MO, USA). The medium was changed every 2–3 days. After 21 days the cells were fixed with 1% paraformaldehyde and stained with von Kossa staining (Bio Optica, Milan, Italy).

### 4.4. Preparation of Cell Vehicles

Four different cell vehicles were tested. Two vehicles, i.e., Ringer Lactate Solution (RLS) and Physiological saline solution 0.9% (PS), are considered nutrient-poor vehicles, while PRP and PPP belong instead to the nutrient-rich vehicles group, providing an environment rich in nutrients and bioactive molecules. RLS and physiological saline solution 0.9% were purchased in sterile bottles (SALF, Bergamo, Italy). PRP and PPP were obtained from autologous venous blood following the protocol described by Suelzu et al. [27]. Fresh blood samples collected in sodium citrate 3.8% *w*/*v* anticoagulant solution (10×) were aliquoted in 15 mL tubes and centrifuged at 190× *g* for 20 min at room temperature (AlC4236A centrifuge ALC, Cologno Monzese, Italy). The erythrocyte fraction was discarded, while the plasma, enriched with platelets, was further centrifuged at 900× *g* for 15 min. The supernatant obtained from this second centrifugation was the plasma poor in platelets (PPP). The platelet pellet obtained following PPP collection was resuspended in a small amount of PPP to perform the platelet count. Finally, a volume of PPP was added to obtain a preparation with a platelet density ranging between 0.8 × 10^9^ and 1 × 10^9^ per milliliter. The latter preparation corresponds to the PRP, that is, Platelet-Rich Plasma. Before using PRP and PPP as vehicles for cellular administration, the coagulation of the two preparations was induced. Since PRP and PPP were collected in sodium citrate, preparing platelet releasate from PRP and serum from PPP was necessary to avoid possible coagulation after cell addition. Coagulation was induced by placing the two vehicles overnight in 10 mL glass tubes and adding 10% calcium gluconate 1000 mg/10 mL (SALF, Bergamo, Italy). The next day the clot was broken with the help of a pipette tip and removed through centrifugation at 900× *g* for 10 min. The recovered liquid supernatant (platelet releasate), no more coagulable, contained all the biological factors released by the breakdown of platelets present in the PRP. The PPP supernatant, being essentially cell-free, contained only the soluble plasma factors. In the present manuscript, the terms sPRP and sPPP indicate, respectively, the supernatant released after PRP and PPP gel formation and centrifugation.

### 4.5. Evaluation of Cell Viability and Vitality

For each donor, cell viability and vitality were evaluated on both fresh cultured MSCs (fMSCs), i.e., actively growing cells detached from culture flasks, and frozen MSCs stored in liquid nitrogen and thawed before use (tMSCs). Both MSC preparations were resuspended in different vehicles simulating a short-time exposure (2 and 4 h), while a long-time exposure was tested on fMSCs (24 h).

#### 4.5.1. Evaluation of Cell Viability and Vitality at 2 and 4 h

Fresh MSC cultures (fMSCs) at passage 3 (P3) were trypsinized upon reaching 70–80% confluence using 0.05% *w*/*v* trypsin solution. The mortality of the cell sample was recorded by Trypan Blue dye assay (Trypan Blue Solution, 0.4% Gibco™, Thermo Fisher Scientific) diluted with cell suspension in a 1:1 ratio, using a Burker camera. The procedure for cell counting is reported in Section 4.5.2 [45]. An aliquot of 1 × 10^6^ cells was then resuspended in 1 mL of each vehicle (PS, RLS, sPPP, sPRP, and DMEM as a control) into 15 mL Falcon tubes. After 2 and 4 h of exposure, cell viability was estimated by Trypan Blue assay, while vitality and replicative capacity were assessed by the MTT (3-(4,5-Dimethylthiazol-2-yl)-2,5-diphenyltetrazolium bromide) (VWR Scientific Avantor, Radnor, OR, USA) colorimetric assay. To this aim, cells exposed to the different vehicles were seeded in a 96-well plate at an initial density of 5000 cells/well (*n* = 6). After 48 h of culture, 20 µL of a 5 mg/mL solution of MTT in phosphate-buffered saline (PBS), pH 7.4, was added. After 4 h, formazan salts were solubilized adding 100 µL of 10% SDS in 0.01 M HCl. Formazan absorbance at 570 nm was recorded after overnight incubation by a Victor Nivo spectrophotometer (Perkin Elmer, Groningen, The Netherlands). The experiment was performed with 4 different cell donors.

The same protocol was repeated with cryopreserved MSCs. Vials containing the frozen cells stored in liquid nitrogen were first thawed at 37 °C and then washed in DMEM added with antibiotics and centrifuged at 190× *g* for 10 min to remove DMSO and FBS. The cell pellet was resuspended and treated as described above for fMSCs. The protocol setup for the short-time experiments with both fresh and frozen cells is resumed in Table 1.

#### 4.5.2. Evaluation of Cell Viability and Vitality at 24 h

Following the results obtained at 2 and 4 h, two vehicles were chosen for the long-time storage experiments on fMSCs: RLS as a nutrient-poor vehicle, and sPRP as a nutrient-rich vehicle. cDMEM was used as a control. Furthermore, the following experiments were performed using two different total cell numbers and volumes, i.e., 1 × 10^6^ cells in 1 mL, and 4 × 10^6^ cells in 4 mL.

In detail, fMSCs at P3 and 70–80% confluent were trypsinized, washed, and resuspended in DMEM to assess cell vitality at time 0 (T0). An aliquot was then resuspended in the two tested vehicles and maintained at room temperature for 24 h. MTT assay was performed as described in the previous paragraph. For the analysis of cell growth, AD-MSCs collected after 24 h of storage were seeded in a 6-wells plate at 6000 cells/cm^2^, incubated at 37° and 5% CO_2_ in cDMEM for 96 h, then detached with trypsin and counted in a Burker camera following incubation with Trypan blue (Trypan Blue Solution, 0.4% Gibco™, Thermo Fisher Scientific). The same parameters were evaluated on cultured non-treated cells as a positive control. To evaluate cell number, after trypsinization, 0.2 mL of the cell suspension was mixed with 0.5 mL of 0.4% Trypan blue and 0.3 mL of PBS. After 5 min, an aliquot of the cell suspension was transferred to the chambers of the Burker camera. Five different 1 mm squares were counted per chamber. Blue-stained cells are considered non-viable cells. The number of viable cells per mL was calculated as follows: average count per square × dilution factor × 10^4^. Two different researchers repeated the count.

The same parameters were evaluated on cultured non-treated cells as a positive control. The protocol setup for the long-time experiments with fresh MSCs is resumed in Table 2.

### 4.6. Gene Expression Analysis

RT-PCR was used for the phenotypical characterization of AD-MSCs [26]. Table 3 shows the specific primers used to evaluate the expression of CD13, CD29, CD31, CD34, CD44, CD45, CD73, CD90, CD105, and OCT-4. The gene expression of fMSCs after long-time storage in sPRP, RLS, and DMEM (4 × 10^6^ in 4 mL) was analyzed through Real-Time RT-PCR and compared with those of cells immediately collected from culture plates. Total RNA extraction was performed from 1 × 10^6^ cell pellet using the NucleoSpin^®^ RNA kit (MACHEREY-NAGEL, GmbH & Co. KG, Hoerdt, France), following the manufacturer’s instructions. Retrotranscription was performed starting from 2 μg of total RNA using the Applied Biosystems™ High-Capacity cDNA Reverse Transcription kit (Applied Biosystems, Cheshire, UK). A total of 1 μL of cDNA solution was used for the qRT-PCR runs. The final composition of the qPCR mixture was 10 μL of AceQ^®^ Universal SYBR Green qPCR Master Mix (Vazyme biotech Co., Ltd., Nanjing, China), 1 μL of cDNA solution, 0.25 μM of specific forward and reverse primers in a final volume of 20 μL. Designed primers (TSG-6) were validated before use by conventional PCR. For this test, 2 μL of cDNA solution was used. The reaction was performed with Bestaq™ DNA Polymerase Kit (Applied Biological Materials Inc., Richmond, BC, Canada) in a final volume of 50 μL. Ethidium bromide was added to samples after PCR (Invitrogen DNA Ladder, Thermo Fisher Scientific), and then samples were loaded onto 1.5% agarose gel to evaluate gene expression. Table 4 shows the specific primers used to evaluate the expression of a set of genes involved in the functional activity of AD-MSCs. The set of genes analyzed included TSG-6, SDF-1, IL-1-Ra, STC-1, COX-2, PGES, CCL-2, and CXCR-4. GAPDH was used as a reference gene. All the primer pairs were previously validated [26,27]. Control experiments were run with negative AD-MSCs markers (CD31, CD45, CD34) using cDNA prepared from canine PBMC (CD31, CD45) and MSCs populations expressing CD34 [26]. One negative control was run for each PCR experiment omitting the cDNA template.

### 4.7. Antioxidant Activity

The FRAP (Ferric Reducing Ability of Plasma) assay was used to evaluate the antioxidant power of the biological fluids, based on the ability of the antioxidants present in the sample to reduce TPTZ (2,4,6-tripyridyl-s-triazine) Fe^3+^ to TPTZ Fe^2+^. The conversion of the ferric ion into a ferrous ion is detectable through a colorimetric reaction of the samples that can be measured by spectrophotometric reading at 595 nm [50]. The preparation of TPTZ was performed just before its use, mixing 25 mL of acetate buffer, 2.5 mL of 2,4,6-Tris(2-pyridyl)-s-triazine (TPTZ) 10 mM in HCl 40 mM and FeCl_3_-6H_2_O solution.

Following the exposure to the different storage vehicles, an aliquot of 5 × 10^5^ cells in 200 μL medium was placed in 96-well plates and treated with Triclosan. Plates were then centrifuged at 400× *g* for 10 min and supernatants were removed. Cold Triton (0.5% *v*/*v*) in PBS containing PMSF, was added to each well to perform cell lysis, which was carried out in an ice bath for 30 min. Fe^3+^ TPTZ was added to 40 μL of cell lysates and the solution was incubated for 30 min at a temperature of 37 °C. The absorbance of Fe^2+^ TPTZ was determined afterwards using the Victor Reader at 595 nm. A calibration curve was set up with known concentrations of FeSO_4_-7H_2_O standard solution to evaluate the ferric-reducing ability of cell lysates.

### 4.8. Statistical Analysis

Each experiment was repeated four times. Data are shown as mean value ± SD.

The statistical differences in the experiments were calculated by ANOVA, using the StatPlusLE 6.7.1 Software (https://www.analystsoft.com/it/products/statplus/ accessed on 1 June 2023). In the presence of a significant difference (*p* < 0.05) the means were subjected to Scheffe and Bonferroni tests for multiple comparisons.

## 5. Conclusions

Storage of therapeutic dosage of MSCs at room temperature can be helpful in different clinical contexts. The coordination and interplay between the needs of the specific clinical case, the needs of the patient and the owner, and the professional activity of the doctor determine time windows in which the cells, prepared for administration, need to be temporarily stored before their practical use. The present work demonstrates that, in these contexts, for short periods, up to at least 4 h, both nutrient-poor and enriched vehicles can adequately perform the function of maintaining the vitality and biological characteristics of the cells. However, variables such as the carrier solution used and the cell concentration should be considered for longer times. Furthermore, the variation in the expression level of genes involved in AD-MSCs functional activity suggests that the storage in different vehicles up to 24 h can affect the therapeutic potential of these cells.

## Figures and Tables

**Table 1:** Short-time storage protocol. A total of 1 × 10^6^ of fresh (fMSCs) and frozen/thawed (tMSCs) MSCs was resuspended in 1 mL of each different vehicle and maintained at room temperature (RT) for 2 and 4 h. Cell viability and vitality were evaluated by Trypan blue dye and MTT assay. RLS: Ringer Lactate solution; PS: physiological saline; sPRP: Platelet Rich Plasma; sPPP: Platelet Pure Plasma.

Cell Preparations	Vehicles	Total Cell Number	Temperature	Time	Tests
fMSCs, tMSCs	sPPP, sPRP, RLS, PS, DMEM (ctrl)	1 × 10^6^ (in 1 mL)	RT	2 h, 4 h	Trypan blue exclusion test, MTT assay

**Table 2:** Long-time AD-MSCs storage protocol. A total of 1 × 10^6^ and 4 × 10^6^ of fresh MSCs (fMSCs) were resuspended, respectively, in 1 mL and 4 mL of each vehicle (sPRP, RLS, DMEM) and maintained at room temperature (RT) for 24 h. Cell growth by direct cell count, Trypan blue exclusion test, and MTT assay were performed to assess cell viability and vitality.

Cell Preparation	Vehicles	Total Cell Number	Temperature	Time	Assay
fMSCs	RLS, sPRP, DMEM (ctrl)	1 × 10^6^ (in 1 mL), 4 × 10^6^ (in 4 mL)	RT	24 h	Trypan blue dye, MTT assay, cell growth

**Table 3:** Primers used for gene expression analysis of phenotypical characterization genes.

Markers	Accession Number	Primers	Amplicon Size	Reference
CD 13	NM_001146034.1	Fw: GGTCCTTACCATCACCTGGC Rv: CCTAAGGCCATCCATCGTCC	335 bp	[26]
CD 29	XM_005616949.1	Fw: AGGATGTTGACGACTGCTGG Rv: ACCTTTGCATTCAGTGTTGTGC	356 bp	[26]
CD 31	XM_848326	Fw: GCCCGAAGTTCACTCTCAAG Rv: CACTCCTTTGACCCACACCT	410 bp	[26]
CD 34	NM_001003341.1	Fw: GAGATCACCCTAACGCCTGG Rv: GGCTCCTTCTCACACAGGAC	383 bp	[26]
CD 44	NM_001197022.1	Fw: CCCATTACCAAAGACCACGA Rv: TTCTCGAGGTTCCGTGTCTC	408 bp	[26]
CD 45	XM_005622282.1	Fw: TGTTTCCAGTTCTGTTTCCCCA Rv: TCAGGTACAAAGCCTTCCCA	432 bp	[26]
CD 73	XM_532221.4	Fw: GATGGGAAAGGCAAGAGGCT Rv: TTCCTGGCATCTTGCTACGG	317 bp	[26]
CD 90	NM_001287129.1	Fw: AAGCCAGGATTGGGGATGTG Rv: TGTGGCAGAGAAAGCTCCTG	285 bp	[26]
CD 105	XM_005625330.4	Fw: GGTTCACTGCATCAACATGG Rv: AAGCTGAAGCGCACATCACC	279 bp	[27]
Oct-4	XM_538830.1	Fw: AAGCCTGCAGAAAGACCTG Rv: GTTCGCTTTCTCTTTCGGGC	286 bp	[26]
GAPDH	NM_001003142.2	Fw: TTCACCACCATGGAGAAGGC Rv: ACTGATACATTGGGGGTGGG	422 bp	[27]

**Table 4:** Primers used for gene expression analysis of functional activity genes.

Markers	Accession Number	Primers	Amplicon Size	Reference
TSG-6	XM_533354.7	Fw: AATCGGATTTCACGTCTGCG Rv: CACCACACTCCTTTGCATGT	182 bp	Generated by: GENE CARDS
SDF-1	NM_001308461.1	Fw: GCCGATTCTTCGAGAGCCAC Rv: TCTGCCATACGCTGTTAGCTT	240 bp	[27]
IL-1-RA	AF216526	Fw: GAAGAGACCTTGCAGGATGC Rv: GACGGGCACCACATCTAACT	141 bp	[46]
STC-1	XM_543238	Fw: CACTTCTCCAACAGATACT Rv: CATGTTGGGCCCAATTTTC	210 bp	[47]
COX-2	NM_001003354.1	Fw: GATCATAAGCGAGGACCAGCTTTC Rv: GGCGCAGTTTATGTTGTCTATCCA	100 bp	[48]
PGES	NM_001122854.1	Fw: GTATTGCCGGAGTGACCAGGA Rv: AGTGCATCTGGGCGATGAAAG	136 bp	[48]
CCL-2	NM_001003297.1	Fw: TCCTCTGCCTGCTGCTCATAG Rv: GCAGCAGGTGACTGGAGAAATAA	86 bp	[49]
CXCR-4	NM_001048026.1	Fw: GAGCGGTTACCATGGAAGAG Rv: CGGTTGAAGTGAGCATTTTCC	108 bp	[29]
GAPDH	NM_001003142.2	Fw: GATGGGCGTGAACCATGAGA Rv: AGTGGTCATGGATGACTTTGGCTA	107 bp	[48]

## Data Availability

Data are contained within the article and Supplementary Materials.

## Supplementary Materials

The supporting information can be downloaded at: https://www.mdpi.com/article/10.3390/ijms25063426/s1.
