# Supplementary material for: Mesenchymal Stromal Cells Derived from Canine Adipose Tissue: Evaluation of the Effect of Different Shipping Vehicles Used for Clinical Administration
Source: Int J Mol Sci. 2024 Mar 18;25(6):3426. doi: 10.3390/ijms25063426 (PMC10970639; doi:10.3390/ijms25063426)
Supplement: Supplementary file 1 [file ijms-25-03426-s001.zip › File S1. materials and producers.docx.pdf]

Materials and Producers

- DMEM - Dulbecco's Modified Eagle's Medium (Thermo Fisher Scientific Inc, Whaltam, USA)
- Labware was from VWR (Avantor, Radnor, USA)
- Fetal Bovine Serum- FBS (Thermo Fisher Scientific Inc, Whaltam, USA)
- Penicillin (Thermo Fisher Scientific Inc, Whaltam, USA)
- Streptomycin (Thermo Fisher Scientific Inc, Whaltam, USA)
- Amphotericin (Thermo Fisher Scientific Inc, Whaltam, USA)
- Trypsin-EDTA (Thermo Fisher Scientific Inc, Whaltam, USA)
- DMSO dimethyl sulfoxide (Thermo Fisher Scientific Inc, Whaltam, USA)
- StemPro Adipogenesis Differentiation Kit (Gibco Thermo Fisher Scientific Inc, Whaltam, USA)
- Stem Pro Chondrogenic Differentiation kit (Gibco Thermo Fisher Scientific Inc, Whaltam, USA)
- Oil Red O stain (Sigma-Aldrich, Inc, St. Louis, USA)
- Alcian blue (Sigma-Aldrich, Inc, St. Louis, USA)
- Dexamethasone (Sigma-Aldrich, Inc, St. Louis, USA)
- glycerophosphate (Sigma-Aldrich, Inc, St. Louis, USA)
- ascorbic acid (Sigma-Aldrich, Inc, St. Louis, USA)
- Paraformaldehyde (Bio Optica, Milan, Italy)
- von Kossa staining (Bio Optica, Milan, Italy)
- Ringer Lactate Solution (RLS) (SALF, Bergamo, Italy)
- Physiological saline solution 0,9% (SALF, Bergamo, Italy)
- AlC4236A centrifuge (ALC, Italy)
- calcium gluconate (SALF, Bergamo, Italy)
- sodium citrate (Thermo Fisher Scientific Inc, Whaltam, USA)
- Victor Nivo spectrophotometer (Perkin Elmer, Groningen, The Netherlands)
- MTT (3- (4,5-Dimethylthiazol-2 -yl) -2,5-diphenyltetrazolium bromide) (VWR Scientific Avantor, Radnor, USA)
- SDS in 0.01 M HCl (Thermo Fisher Scientific Inc, Whaltam, USA)
- Trypan Blue Solution 0.4% (Gibco™, Thermo Fisher Scientific)
- NucleoSpin® RNA kit (MACHEREY-NAGEL, GmbH & Co. KG)
- Applied Biosystems™ High-Capacity cDNA Reverse Transcription kit (Applied Biosystems, Cheshire, UK)
- AceQ® Universal SYBR Green qPCR Master Mix (Vazyme biotech co., ltd)
- Bestaq™ DNA Polymerase Kit (Applied Biological Materials Inc.,|Richmond, Canada)
- Ethidium bromide (Invitrogen DNA Ladder, Thermo Fisher Scientific – US)
- RT-PCR PRIMERS (Macrogen Europe Meibergdreef 57 1105 BA, Amsterdam, The Netherlands) and (Eurofins Genomics, Germany GmbH Anzinger Str. 7a 85560 Ebersberg, Germany)
