# Supplementary figures and images for: Mesenchymal Stromal Cells Derived from Canine Adipose Tissue: Evaluation of the Effect of Different Shipping Vehicles Used for Clinical Administration
Source: Int J Mol Sci. 2024 Mar 18;25(6):3426. doi: 10.3390/ijms25063426 (PMC10970639; doi:10.3390/ijms25063426)

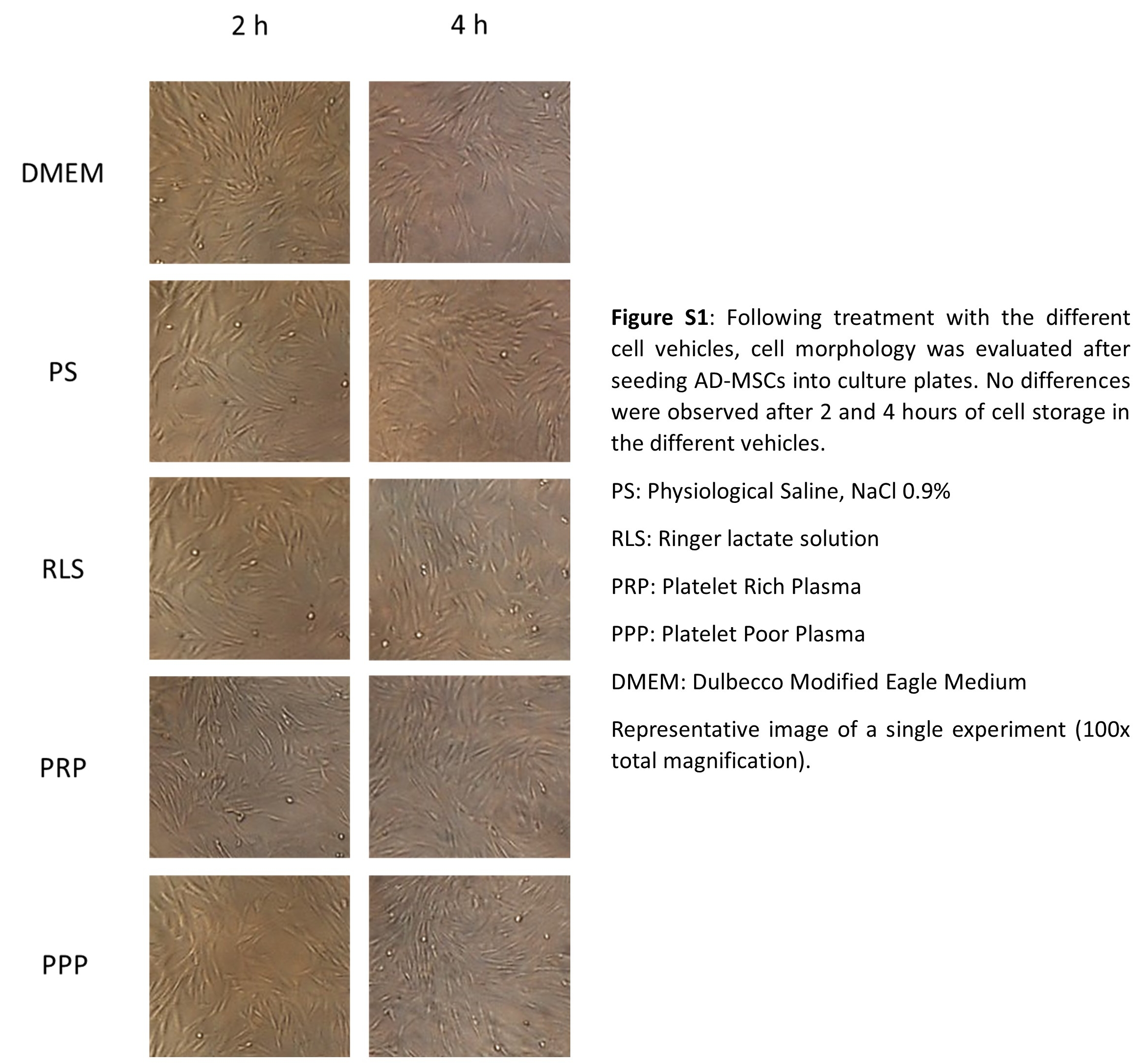

Supplement: Supplementary file 1 [file ijms-25-03426-s001.zip › Figure S1.jpg]

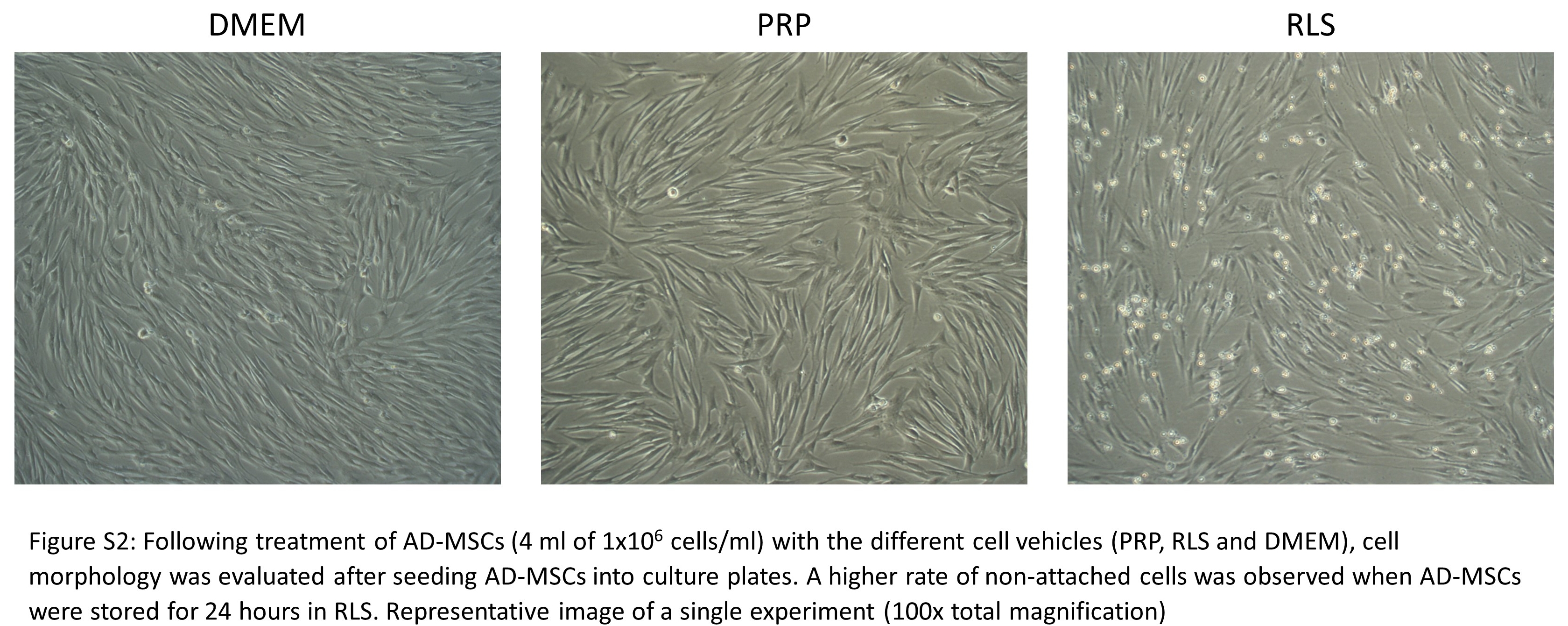

Supplement: Supplementary file 1 [file ijms-25-03426-s001.zip › Figure S2.jpg]
